# Training of Awareness in ADHD: Leveraging Metacognition

**DOI:** 10.20900/jpbs.20240006

**Published:** 2024-10-09

**Authors:** Agatha Lenartowicz, Brett DeSchepper, Gregory V. Simpson

**Affiliations:** 1 Department of Psychiatry & Biobehavioral Sciences, OneMind Staglin Center for Cognitive Neuroscience, Semel Institute for Neuroscience and Human Behavior, University of California Los Angeles, Los Angeles, CA 90095, USA; 2 Think Now Inc., San Francisco, CA 94105, USA

**Keywords:** ADHD, awareness, metacognition, psychosocial, training, non-pharmaceutical

## Abstract

Attention deficit hyperactivity disorder (ADHD) is a disorder that is prevalent in children and adults, with significant impact on life outcomes. Common treatment strategies include a combination of pharmacological and psychosocial interventions which have recognized limits to their effectiveness. Consequently, there exists interest in additional non-pharmacological interventions. In the current minireview we aim to complement existing surveys by focusing on a complementary approach, namely rooted in metacognition or the training of awareness. We review programs that incorporate metacognitive training of awareness in skill-training, psychosocial interventions, and mindfulness, and discuss existing assessments of metacognitive ability in ADHD. Existing data suggest that metacognitive approaches have potential in supporting symptom management in ADHD, with gains in objective assessments in near and far transfer tasks in educational research and high satisfaction from parents. Further research is warranted in assessment of the relative contribution of metacognitive elements relative to other treatment components, objective assessments of outcomes in psychosocial interventions, and efficacy in adult interventions.

## INTRODUCTION

Attention deficit and hyperactivity disorder (ADHD) is characterized by symptoms of inattention, impulsivity and hyperactivity [1] that have significant consequences on multiple aspects of life including social, educational, and professional [2-5]. Its prevalence rates are around 3% [2,6] for adults and 5%–7% for children [2,7], with 60% persistence into adulthood [8]. Current treatments include psychosocial and pharmacological interventions [9,10]. Medications (including stimulants and non-stimulants) have a high-reported efficacy in reducing symptoms [11,12]. However, they are accompanied by mixed concerns about side-effects [13-15], limitations in cases of substance abuse co-morbidity [16], and issues of adherence [17-19]. Thus, non-pharmacological alternative or complementary interventions continue to be of significant interest as evidenced by numerous reviews [20-24], with greatest empirical evidence amassed in support of psychosocial interventions [20,23,25], and increasingly also neurofeedback [26,27] and physical exercise [23,24,28,29], with data also emerging to inform efficacy of digital [22] and mindfulness-based [30] interventions.

In the current minireview we aim to complement these existing surveys by focusing on another key element that has emerged in non-pharmacological interventions, namely metacognition or the training of awareness. Metacognition refers to knowledge of one’s cognition (i.e., self-knowledge or self-awareness of one’s cognitive and learning processes), as well as related metacognitive skills and experiences [31]. It includes regulation of cognition, encapsulating planning, monitoring and evaluating behavior, and translating into predicting, checking and adjusting of one’s cognitive processes [32]. Critically, such self-regulation has a demonstrated role in improving learning outcomes in the educational domain [33-37], with a proposed role in supporting far-transfer [38], and a similarly recognized role in therapeutic action in psychiatry [39,40], potentially by supporting the self-awareness of and action on maladaptive neurobehavioral processes rather than content [41]. These elements of metacognitive training may render it particularly appropriate to addressing a key challenge in ADHD, the recognized heterogeneity in etiology, symptoms, cognitive deficits, and medication responses in this population [2,42-48], by targeting self-regulation on an individual basis [21]. The goal of this minireview is to present a current survey of published interventions in ADHD that feature metacognitive training.

### Metacognitive Training in ADHD

To identify published works that include metacognition in ADHD interventions, we conducted three searches using Web-of-Science (Clarivate), PubMed and Google Scholar literature databases. The search was conducted with key words “metacognition”, “awareness” and “ADHD” included in the topic or abstract field. We contacted original authors for works not available through library resources, and identified additional articles based on current reviews. We also conducted a future search (i.e., articles that cited found articles) for a more complete search. We eliminated works that reported on “awareness of ADHD” in educators, medical practitioners or within broader society (i.e., were not empirical intervention studies). This search produced a total of 17 empirical works (Tables 1 & 2) on the role of metacognitive training in treatments for ADHD that could be classified into categories based on the complementary treatment goal. These include (1) skill training (e.g., executive function or working memory) most prevalent in efforts targeting educational outcomes; (2) psychosocial interventions (e.g., cognitive behavioral therapy) dominant in clinical settings and which include parental training; and (3) existing mindfulness interventions adapted to ADHD. We review each category in turn, with additional consideration of key factors such as metacognitive ability.

### Metacognition in skill training

#### Educational research paradigms

A systematic effort to implement metacognitive awareness training into ADHD treatment has been documented within educational research, where the role of metacognition in self-regulated learning has demonstrated value for improving educational outcomes [35,36], especially in promoting far transfer [34,38]. One of the first such training programs was developed in 2013 by Garcia-Madruga et al. [49] to target reading comprehension in a small sample of 8–9-year-olds (15 in experimental condition and 16 in control). Motivated by a need to improve far-transfer effects, they included a broad range of activities, natural learning contexts and progressive increases in difficulty, along with iterative refinements of training materials. They reported gains in both reading comprehension, as well as visuo spatial and working memory assessments relative to a control (*d* = 0.25–0.79). Subsequent studies [50-52] elaborated on the proposed 60 min training sessions to bookend a series of activities targeting a specific skill, with a metacognitive introduction at the start and metacognitive reflection at the end. Such training sessions would then be delivered to groups of students in a classroom setting, for a total of 1–2 sessions/week, over 8 to 11 weeks. Statistically significant gains from metacognitive training across these studies were demonstrated for training of problem solving [52] as well as reading & listening comprehension [50,51]. Notably the gains were replicated in larger sample sizes (e.g., *n* = 64–69/training group [51,52]), in a cross-over design [52] (with all groups receiving treatment at some point in the study) and relative to both active (non-metacognitive training program) and passive (standard school experience) control groups [50,51].

This effort was followed by three studies [53-55] where a modification of the training program was developed to target working memory and executive function abilities in kids with ADHD (Age 5.2–8.5 years, Table 1). Across the studies the same metacognitive training session structure (i.e., metacognitive strategy and reflection with feedback book-ending a topical training activity) was applied to support training of working memory [53-55], as well as inhibition [55]. In these studies, groups of kids with and without ADHD were assigned into both training and control groups (13–20 individuals per group). In the control group kids participated in typical school activity in lieu of intervening training but had identical pre and post study assessment. Moderate significant gains were observed in objective assessments of working memory [53] and inhibition [55] in the training group for kids with ADHD, but not in the non-training group. Additionally, training gains were not significant for most measures for typically developing peers. Notably, assessment of transfer effects [54] showed that following metacognitive training of working memory, gains were also observed in performance on inhibition and attention tasks, and nonverbal reasoning, with gains significant across several measures in a 1 month follow up. Finally, a study by Pisacco et al. [56] in older children (Age = 13.1 years, *n* = 47), employing two different metacognitive programs of similar dosage structure, showed gains in written expression that persisted in a 3-month follow up. Interestingly, across these four studies, gains were inconsistent in parent/teacher self-report measures of ADHD symptoms, which may suggest that the extent of transfer is limited to cognitive performance or that the self-report symptoms capture a different dimension of behavior than the performance assessments.

#### Role of skills versus metacognition

An important limitation of the reviewed metacognitive training is that, in application to ADHD, the studies did not separate the relative contributions of skill-specific training (e.g., working memory activities) versus the metacognitive elements to the gains in outcomes. However, some evidence exists to suggest that the metacognitive elements may have a key role. An earlier study using the same session structure for training of reading comprehension in typically developing children (9–11 years), did include an active control group (*n* = 57) who received the same reading training program as the experimental group (*n* = 45), but without the metacognitive elements [51]. They reported gains over the active control group with moderate to large effects sizes (*d* = 0.6–1.2) and sustained gains to reading in an 8 month follow up. Similarly, Zheng et al. [57] added about 10 minutes of metacognitive reflection at the end of an astronomy science class in 5th grade students with ADHD, and showed that after 15 classes and in a 2-week follow-up, gains in scientific knowledge (math, spatial) and learning motivation had greater learning-effect sizes in kids with the added reflection (*n* = 49) than those without (*n* = 48). Finally, Kajka & Kulik in a similar age group (Age = 10.4 years), showed that training only on metacognitive thinking via the so-called mind maps learning strategy which uses visual notes to organize idea development, after 25 training sessions using a variety of idea topics, significantly increased verbal fluency in kids with ADHD [58]. Thus, some evidence exists to support learning gains in kids with ADHD when metacognitive reflection is systematically incorporated into skill training.

In contrast, several studies have implemented ecological training programs of executive function skills, but with emphasis on skill acquisition more than metacognitive reflection. Such studies report gains in self-reported symptoms, but lack gains on objective assessments [61], consistency in gains across assessments [59,60] or persistence of gains in follow up [59]. Namely, Paananen et al. [59] compared an established interactive executive function coaching system (Maltti) in school age children with ADHD (Age = 9.5 years, *n* = 77) in Finland, and after a weekly session for the duration of a school-semester, reported improvements in cognitive control (i.e., inattentive) symptoms but not hyperactive symptoms, and the follow-up gains did not differ from those in a waitlist control group (*n* = 77). Tamm et al. [60] examined a commercial program (*Pay Attention!* [62]), designed to train sustained, selective, alternating, and divided attention via flexible coaching sessions. Following 16 bi-weekly sessions (Age = 9.1 years, *n* = 54), as compared to a waitlist group (Age = 9.5 years, *n* = 51) significant improvement was observed in about half of the objective Test of Everyday Attention for Children sub-scales, in parent and clinician, but not teacher reported behavior scores, and none but one of the objective neuropsychological tests. Finally, Qian et al. [61] tested Dawson & Guare’s 2010 [63] ecological executive skill straining program (Age = 8.3 years, *n* = 38 versus waitlist Age = 7.8 years, *n* = 30) to report, after 12 weekly 60 min sessions, significant improvements across self-reported symptoms and about half of self-reported executive function scales but not on objective assessments (e.g., Stroop Color-Word and Trail-Making Tests). As such, benefits from ecological training of executive function appear inconsistent, and in some cases driven by compensatory gains in participants with low baseline scores [64].

### Metacognition in psychosocial interventions

#### Parental training

A relevant factor in efficacy of metacognitive training in children may include metacognitive support from parents. Three recent studies tested this hypothesis (Table 2), implementing 8–10 weeks of parental training with a 1–2-hour weekly session that explicitly included elements of metacognitive awareness, and reported significant benefits in measures of child-parent relationship, parental satisfaction, as well as in daily routine efficiency and ADHD symptoms [65-67]. Namely, Frisch et al. [65] enrolled 39 families (vs 33 waitlist families in cross-over design) to participate in a parental occupational executive training program (POET, 8 weekly sessions), which combined training in what executive functions are and how symptoms arise (e.g., impulsivity and delayed planning), and occupational solutions. Parents were explicitly instructed to raise awareness in children through simple explanations of the learned concepts. Benefits were significant in parent-reported ability to complete daily routines (e.g., morning-evening) and impulsivity (but not inattentive) symptoms, maintained in an 8–12-week follow-up. Second, Shah et al. [67] designed an adaptation of existing parent training approaches founded in operant conditioning and social learning theory (e.g., contingency management and behavior management techniques) [68-70], to incorporate values culturally appropriate in India. Doing so involved adding a biological perspective, home role-playing tasks to improve generalization, incorporated extended family in line with collectivistic values, and spiritual methods like yoga and meditation to reduce stress, that together may be viewed as adding metacognitive awareness into the training. The 10-week training program (*n* = 41) produced significant benefits in symptoms (inattentive & hyperactive) and school performance & classroom behavior, with benefits comparable regardless of whether the child was on medication, thus highlighting potential complementary value of benefits. It is notable that the benefits were reliable despite average completion rate being 6.89/10 sessions. Finally, Hahn-Markowitz et al. [66] reported significant increases in parent self-efficacy across 5/7 measured constructs, following a 10-week cognitive functional training program that uses metacognitive learning of executive strategies (e.g., stop, do/persist, check, plan) (*n* = 50/group, cross-over design). This result is consistent with parent-reported increases in awareness, reduction in guilt/blame and stress, and improvement in parent-child relationships & self-reliance, reported by Shah et al. [67] and others [71,72].

It is notable that the trainings did not improve inattentive symptoms in one study [65], and anxiety/depression scores in another [67] (also see CBT training Table 2), perhaps suggesting that parent training is less effective at scaffolding cognition or internalizing behaviors, than social or externalizing behaviors. If so, it may serve as a complement to programs that target metacognitive and/or skill training in children, as described in the previous section. Though research to address this question is lacking, relevant results were published by Tamm et al. [72] who, in a feasibility study, incorporated parent training within a combined metacognitive and executive skill training program in a small sample of children with combined-type ADHD (Age = 5.2 years, *n* = 24). The program included 60 min group training sessions, for 8 weeks, in which naturalistic activities were used to teach executive function skills, along with metacognitive reflection on skill understanding and performance strategies. Parents met in groups for analogous training that included metacognitive reflection on situations in which skills may be needed, and practiced implementation to be repeated in the home. Significant improvements were reported in 3/7 objective assessments of visual attention (NEPSY [77]), and in self-reported symptoms and scales of executive function. However, as a control group was not included, replications are warranted. Notably, and consistent with reports by others [67], the parent satisfaction ratings were high, with average rating of >5 on a 7-point Likert scale and 96% completion rate. In sum, this work supports the notion that parent-child metacognitive training has the potential to produce a mutualistic relationship that can support skill training in ADHD, while highlighting the need for further research with emphasis on objective outcomes and appropriate controls.

#### CBT in Adult ADHD

In the case of adult ADHD, reports of treatments targeting metacognitive awareness are fewer and nested within the context of cognitive behavioral training programs (CBT). It is notable that some have debated whether such approaches truly address metacognition [78]. However, as articulated by Moritz et al. [79], it is difficult to dispute that CBT approaches, by nature of including self-reflection and experience of targeted cognitions or behaviors, satisfy prevalent definitions of metacognition [31] by way of fostering metacognitive knowledge and experience. Thus, the debate may be in terms of degree to which a given CBT program incorporates metacognitive awareness, though operationalizing which programs do or do not include metacognitive elements is a clear challenge. Here we review programs that explicitly discuss metacognitive awareness or strategy or assess metacognition in outcomes.

In ADHD, CBT programs that meet these criteria include Solanto et al. [73-75] and Safren et al. [76] (Table 2). Solanto et al. [73] developed a 12-week (2hr/week) CBT that focused on time management, organization, and planning skills, and integrated discussion of and reflection on cognitive-behavioral strategies to promote metacognitive awareness. Compared to supportive therapy (*n* = 43) that lacked such discussions, the treatment group (*n* = 45) improved on Adult ADHD Investigator Symptoms Rating Scale [80] assessment of inattention, Conners inattention and memory scales, as well as time management sub-scales and self-rating executive functions scores. Improvement was significantly larger than in the supportive therapy group (*p* < 0.05). The treatment group also showed lower attrition (16% vs 37%) and higher responder rates (42.2% vs 12%) than the supportive therapy group [73]. A randomized controlled trial (RCT) of the program was subsequently performed in older adults with analogous results [74]. More recently the program was adapted to academic environment needs of college students with positive results in a feasibility trial that did not include a control group [75], including replication of low attrition (17%) and 67% completion rate for home work exercises, previously shown to improve treatment outcome [73]. A comparable CBT program was described by Safren et al. [76,81] which focused on the training of “compensatory strategies in organizing and planning, coping with distractibility and enhancing optimal thinking strategies”, reporting decreases in ADHD symptom severity (effect size = 1.2) and clinical global impression (effect size = 1.4), and 56% responder rate in the treatment group (*n* = 16, medication + CBT) when compared to a group on only pharmacological treatment (*n* = 15, responder rate 13%). In sum, the existing data suggest positive effects from CBT that incorporates metacognitive awareness strategies in adults with ADHD, though the small number of adequately powered and controlled RCTs, and lack of objective measures in this domain warrant further studies.

### Metacognition in mindfulness training

An additional domain of treatment in ADHD that features elements of metacognition is that of mindfulness and meditation training. Mindfulness meditation techniques aim to foster awareness to one’s present experience, through silent sitting, walking or purposeful attention to daily activities. Such programs thus engage intentional self-regulation of attention [82,83], which falls within the definition of metacognitive awareness. Unlike the context of skill training or psychosocial interventions, mindfulness training does not target a specific ADHD symptom or skill. However, ADHD treatments based on mindfulness meditation activities have been developed, in part motivated by proposed associations between mindfulness and neural systems of attention [82,84], and could offer a valuable assessment of whether generalized awareness training is sufficient to improve ADHD skills over skill training. Numerous systematic reviews and meta-analysis have been published on the efficacy of mindfulness-based training programs in ADHD [30,85-91], and thus we focus here on summarizing key trends. Across meta-analyses, mindfulness and meditation training programs report significant reductions in ADHD symptoms (Hedges’ g = −0.17 to 4.03 [85] with most studies in the medium to large effect size range), with comparable, large effect sizes across self-reported and other-reported symptoms [90]. In three meta-analyses, the effect size was reported as larger in adults than children [30,88,90]. However, assessment of evidence quality suggest high bias is prevalent across reviewed studies [30,87,89,91], leading some to conclude that despite significant effects the evidence is methodologically insufficient to support meditation-based therapies for ADHD [30]. As such, data on the efficacy of mindfulness in ADHD is inconclusive, warranting further study.

### Role of metacognitive ability

Finally, it is of note that metacognitive training, in any context, relies on metacognitive abilities. Thus, it is pertinent to ask if metacognitive training in ADHD bestows benefits via the training of metacognitive ability itself or if it scaffolds skill training, in line with the supportive role of metacognition in self-regulated learning (e.g., by way of engaging executive functions like planning [92] or by increasing resilience to anxiety through motivation [93]). One way to address this question is to ask, is metacognitive ability preserved in ADHD or does it differ, along with executive function performance with which it is intricately intertwined [94,95]. At first glance the findings are mixed. One recent study of 7–14-year-old children (*n* = 60 with ADHD), reported that scores on the Metacognitive Awareness Inventory were significantly lower in kids with ADHD than those without and correlated negatively with symptoms of ADHD as well as depression and anxiety. Yet, two other studies, report no group differences in metacognition. Slobodin et al. [96], also in 7–10 year old children with ADHD (*n* = 190), found that self-ratings of inattention correlated with objective performance scores on a sustained attention task, suggesting accurate self-awareness. Similarly, in adults, Butzbach et al. [97] (*n* = 47 ADHD, *n* = 47 non-ADHD) replicated performance deficits in attention in ADHD but did not find group differences in ability to estimate own performance in executive function, and memory. A small effect-size for over-estimation of attention was reported in participants with ADHD.

Thus, a global deficit in metacognition in ADHD is not supported by existing data. Further clarity on the discrepant findings may be gained from studies that use componential or multi-dimensional assessments of metacognition. For instance, Pezzica et al. [98] used a Children’s Awareness of Attention through Drawing tool to assess metacognition in 92 primary school age kids (*n* = 45 with ADHD, 5–11 years). They coded visual representations of attentive and inattentive constructs in children across five dimensions of awareness (behavioral, pragmatic, cognitive, social, and emotional). They found that children with and without ADHD had similar representations of attention versus inattention suggesting similar metacognitive constructs. They differed however in ability to organize educational tools and in the emotional state associated with school, with more negative emotion in visual representations of school in children with ADHD. This difference emerged with age (8–11 > 5–8 years). This finding supported the conclusion that in children with ADHD, negative feelings around attention increase with age, potentially through social learning and self-awareness around the deficits. This conclusion is consistent with the results of an adult study (*n* = 40 ADHD, *n* = 42 non-ADHD), in which a sub-component analysis of responses on the Metacognitions Questionnaire-30 (MCQ-30) revealed that only the score on the confidence subscale of the MCQ-30 predicted inattentive scale scores, and there were no group differences in a score of general cognitive consciousness. These studies suggest that metacognition in individuals with and without ADHD is not reliably different, and thus metacognitive training may exert positive effects through scaffolding of executive function or motivational processes needed for behavioral management.

## OUTSTANDING QUESTIONS & CONCLUSIONS

The reviewed data highlight both that metacognitive approaches have potential in supporting symptom management in ADHD, and that much research in this domain is needed and warranted. The premise that metacognition may serve to support symptom management is bolstered by evidence of metacognitive awareness in individuals with ADHD. Educational context, where research reports are most numerous and report positive effects in objective assessments and near and far-transfer following training, offers a valuable foundation for future research designs that include objective quantifiable outcomes. Notable methodological gaps include refinements on the relative contributions of metacognitive elements versus skill training in combined approaches, continued development of objective assessments, especially in psychosocial approaches such as parental training and CBT (*cf.,* Table 2), and systematic assessment of metacognitive training in adult ADHD, which is sparse. The need for objective outcomes is particularly important for reducing attrition rates in self-report of symptoms, which reduces the impact of long-term follow up studies [65,67].

Furthermore, given increasing notice of gender differences in ADHD symptoms and outcomes [99-102], the role of gender in effects of metacognitive training in this population is an important understudied research goal. In the reviewed studies, a bias towards males was present across study samples (Table 1 mean = 33% females, Table 2 mean = 40% females). Though 5 of the 17 reviewed studies included gender as a covariate or moderating variable and found no significant effects [60,61,65,66,73], the role of gender will require additional systematic study especially in larger samples. Similarly, the effect of age on efficacy of metacognitive training warrants further study. In the reviewed studies significant results were reported in children as young as five, though with the scaffolding provided by the educator or parent potentially playing a significant part. Finally, it is notable that metacognition may play an important role in shaping other existing alternative treatments such as neurofeedback or some digital interventions (e.g., brain training), that do not currently involve metacognition and thus are not considered in the reviewed works. In conclusion, metacognitive training, with focus on awareness and self-regulation, both shows clear potential to support symptom management and warrants research across several domains, thus defining a distinct frontier in the field of ADHD treatments.

## Figures and Tables

**Table 1.** Metacognitive & skill training in ADHD.

Author	Year	Mean Age	Sample Size T/C_ADHD_ T/C_TD_	Training				Outcome	
				Skill	MC	Dose M/D/W	Control	Subjective (Scales)	Objective (Tasks)
Educational Context & Metacognitive Training									
Re et al. [55]	2015	5.4	13*(5f)*/13*(4f)* 13*(8f)*/13*(7f)*	EF	Explicit in skill lesson	60/2/9	School Activities	Symptoms	Inhibition*, Impulsivity*, WM
Capodieci et al. [53]	2018	5.5	18*(4f)*/16*(2f)* 20*(10f)*/20*(8f)*	WM	Explicit in skill lesson	60/2/8	School Activities	Symptoms	Inhibition*, Impulsivity*, WM*
Capodieci et al. [54]	2019	7.2	12*(3f)*/− 15*(3f)*/−	WM	Explicit in skill lesson	60/1/8	-	Symptoms^†^	visual WM^††^, visual-SA^††^, auditory-SA, Inhibition^††^, Impulsivity^††^, MR^††^
Pisacco et al. [56]	2018	13.1	47*(13f)*/− −/−	Writing & WM	Explicit in skill lesson	45/2/13	-	Hyperactivity^*^, Inattention	WM, Written Expression^**^
Zheng et al. [57]	2021	9.8	49*(24f)*/48*(24f)* −/−	Science	10 min post skill lesson	45/15/−	Omit MC training	Metacognition^*^, Motivation^*^	Math^†^, Spatial^†^
Kajka & Kulik [58]	2023	10.4	(15*(2f)*/15*(3f)*)/ 15*(2f)* −/−	-	(Sketchnote /Mindmap)	60/2/13	Waitlist	-	Verbal Fluency^*^
Ecological Skill Training									
Paananen et al. [59]	2022	9.5	71/77 −/−	EF	-	60/20/−	Waitlist	Inattention Symptoms^*^	-
Tamm et al. [60]	2013	9.3	54*(18f)*/51*(16f)* −/−	Attention	-	30/2/8	Waitlist	Symptoms (clinician/parent)^*^, Symptoms (teacher)	TEA-Ch Attention (4/9 scores^*^)
Qian et al. [61]	2017	8.1	38*(6f)*/30*(8f)* −/−	EF	-	60/1/12	Waitlist	EF^*^, Symptoms^*^	Trails, Stroop

Notes. * = significant in ADHD training group, ** = significant in ADHD training group at follow-up, ^†^ = significant in all training groups (ADHD and non-ADHD), ^††^ = significant in all training groups at follow-up, T/C = training/control (a “−” indicates that this group was not included in study), *(f)* = number of females in sample, TD = typically developing, MC = metacognitive training, M/D/W = minutes/days/weeks, EF = executive function, WM = working memory, MR = matrix reasoning, SA = selective attention, TEA-Ch = test of everyday attention—child.

**Table 2.** Metacognitive elements in psychosocial interventions in ADHD.

Author	Year	Mean Age	Sample Size T/C_ADHD_ T/C_TD_	Training				Outcome	
				Program	MC	Dose M/D/W	Control	Subjective (Scales)	Objective (Tasks)
Parental Training									
Frisch et al. [65]	2023	5.4	39/33 *(17f total)* −/−	Parental Occupational Executive Training	Raising awareness via examples	−/1/8	Waitlist	Impulsivity^**^, Inattention, Parent-reported ability to complete daily routines^**^	-
Shah et al. [67]	2021	9.6	36*(2f)*/− −/−	Parent Skill Training Intervention	Cultural adaptations (discuss biological perspective, role-playing, yoga, meditation)	90/1/10	-	Inattention^*^, Hyperactivity^*^, School Performance^*^, Depression/Anxiety	-
Hahn-Markowitz et al. [66]	2018	8.5	50*(17f)*/49*(14f)* −/−	Cognitive-Functional Intervention	Metacognitive learning of executive strategies	60/1/10	Waitlist (Cross-Over)	Self-reported parenting efficacy^**^	-
Tamm et al. [72]	2014	5.2	24*(6f)*/− −/−	EF skill training + parental training	Explicit in child lessons + parent as teacher	60/1/8	-	Inattention^*^, Hyperactivity^*^, EF^*^, MC index^*^	NEPSY (following directions^*^, WM^*^, control efficiency^*^, visual SA, MR, inhibition, concurrent efficiency)
Adult CBT									
Solanto et al. [73]	2010	41.7	45*(32f)*/43*(26f)* −/−	CBT (time, organization, planning)	Explicitly present, discuss, and train the application of executive strategies	2/1/12	Supportive therapy	Inattention^*^, EF^*^, On-Time Management & Planning^*^, Depression/Anxiety, Self-Esteem	-
Solanto et al. [74]	2018	35.3 & 56.2	29/29 (younger, *41f*) 12/14 (older, *17f*) −/−	As above	As above (tested in older adults)	2/1/12	Supportive therapy	Inattention^*^, EF^*^, On-Time Management & Planning^*^, MC-Index^*(older only)^	-
Solanto et al. [75]	2021	22.6	18*(10f)*/− −/−	As above	As above (adapted to academic tasks)	2/1/12	-	Inattention^*^, EF^*^, On-Time Management & Planning^*^, Depression/Anxiety, Worry^*^, Learning Study Skills^*^	-
Safren et al. [76]	2005	45.5	16/25 −/−	Medication + CBT (organizing, planning, time, adapting thinking)	Practice of compensatory strategies	8 sessions in 3 modules	Medication alone	ADHD symptoms^*^, CGI^*^, Anxiety^*^, Depression^*^	-

Notes. * significant in ADHD training group, ** significant in ADHD training group at follow-up, ^†^ significant in all training groups (ADHD and non-ADHD), ^††^ significant in all training groups at follow-up, T/C = training/control (a “−” indicates that this group was not included in study), TD = typically developing, *(f)* = number of females in sample, MC = metacognitive training, M/D/W = minutes/days/weeks, EF = executive function, MR = matrix reasoning, SA = selective attention, CGI = clinical global impression.

## Data Availability

No data were generated from the study.

## References

[1] Association AP. Desk reference to the diagnostic criteria from DSM-5-TR. Washington (US): American Psychiatric Association Publishing; 2022.

[2] Song P, Zha M, Yang Q, Zhang Y, Li X, Rudan I. The prevalence of adult attention-deficit hyperactivity disorder: A global systematic review and meta-analysis. J Glob Health. 2021;11:04009. 33692893 10.7189/jogh.11.04009 PMC7916320

[3] Kuriyan AB, Pelham WE, Molina BS, Waschbusch DA, Gnagy EM, Sibley MH, Young adult educational and vocational outcomes of children diagnosed with ADHD. J Abnorm Child Psychol. 2013;41(1):27–41. 22752720 10.1007/s10802-012-9658-z PMC3505256

[4] Biederman J, Monuteaux MC, Doyle AE, Seidman LJ, Wilens TE, Ferrero F, Impact of executive function deficits and attention-deficit/hyperactivity disorder (ADHD) on academic outcomes in children. J Consult Clin Psychol. 2004;72(5):757–66. 15482034 10.1037/0022-006X.72.5.757

[5] Biederman J, Petty C, Fried R, Fontanella J, Doyle AE, Seidman LJ, Impact of psychometrically defined deficits of executive functioning in adults with attention deficit hyperactivity disorder. Am J Psychiatry. 2006;163(10):1730–8. 17012683 10.1176/ajp.2006.163.10.1730

[6] Fayyad J, Sampson NA, Hwang I, Adamowski T, Aguilar-Gaxiola S, Al-Hamzawi A, The descriptive epidemiology of DSM-IV Adult ADHD in the World Health Organization World Mental Health Surveys. Atten Defic Hyperact Disord. 2017;9(1):47–65. 27866355 10.1007/s12402-016-0208-3 PMC5325787

[7] Sayal K, Prasad V, Daley D, Ford T, Coghill D. ADHD in children and young people: prevalence, care pathways, and service provision. Lancet Psychiatry. 2018;5(2):175–86. 29033005 10.1016/S2215-0366(17)30167-0

[8] Sibley MH, Swanson JM, Arnold LE, Hechtman LT, Owens EB, Stehli A, Defining ADHD symptom persistence in adulthood: optimizing sensitivity and specificity. J Child Psychol Psychiatry. 2017;58(6):655–62. 27642116 10.1111/jcpp.12620 PMC5809153

[9] Cortese S, Asherson P, Sonuga-Barke E, Banaschewski T, Brandeis D, Buitelaar J, ADHD management during the COVID-19 pandemic: guidance from the European ADHD Guidelines Group. Lancet Child Adolesc Health. 2020;4(6):412–4. 32311314 10.1016/S2352-4642(20)30110-3 PMC7164889

[10] Mechler K, Banaschewski T, Hohmann S, Hage A. Evidence-based pharmacological treatment options for ADHD in children and adolescents. Pharmacol Ther. 2022;230:107940. 34174276 10.1016/j.pharmthera.2021.107940

[11] Faraone SV, Buitelaar J. Comparing the efficacy of stimulants for ADHD in children and adolescents using meta-analysis. Eur Child Adolesc Psychiatry. 2010;19(4):353–64. 19763664 10.1007/s00787-009-0054-3

[12] Faraone SV, Glatt SJ. A comparison of the efficacy of medications for adult attention-deficit/hyperactivity disorder using meta-analysis of effect sizes. J Clin Psychiatry. 2010;71(6):754–63. 20051220 10.4088/JCP.08m04902pur

[13] Hennissen L, Bakker MJ, Banaschewski T, Carucci S, Coghill D, Danckaerts M, Cardiovascular Effects of Stimulant and Non-Stimulant Medication for Children and Adolescents with ADHD: A Systematic Review and Meta-Analysis of Trials of Methylphenidate, Amphetamines and Atomoxetine. CNS Drugs. 2017;31(3):199–215. 28236285 10.1007/s40263-017-0410-7 PMC5336546

[14] Man KKC, Hage A, Banaschewski T, Inglis SK, Buitelaar J, Carucci S, Long-term safety of methylphenidate in children and adolescents with ADHD: 2-year outcomes of the Attention Deficit Hyperactivity Disorder Drugs Use Chronic Effects (ADDUCE) study. Lancet Psychiatry. 2023;10(5):323–33. 36958362 10.1016/S2215-0366(23)00042-1

[15] Nanda A, Janga LSN, Sambe HG, Yasir M, Man RK, Gogikar A, Adverse Effects of Stimulant Interventions for Attention Deficit Hyperactivity Disorder (ADHD): A Comprehensive Systematic Review. Cureus. 2023;15(9):e45995. 37900465 10.7759/cureus.45995 PMC10601982

[16] Barbuti M, Maiello M, Spera V, Pallucchini A, Brancati GE, Maremmani AGI, Challenges of Treating ADHD with Comorbid Substance Use Disorder: Considerations for the Clinician. J Clin Med. 2023;12(9):3096. 37176536 10.3390/jcm12093096 PMC10179386

[17] Faraone SV, Biederman J, Zimmerman B. An analysis of patient adherence to treatment during a 1-year, open-label study of OROS methylphenidate in children with ADHD. J Atten Disord. 2007;11(2):157–66. 17494833 10.1177/1087054706295663

[18] Adler LD, Nierenberg AA. Review of medication adherence in children and adults with ADHD. Postgrad Med. 2010;122(1):184–91. 20107302 10.3810/pgm.2010.01.2112

[19] Lv YB, Cheng W, Wang MH, Wang XM, Hu YL, Lv LQ. Effect of non-pharmacological treatment on the full recovery of social functioning in patients with attention deficit hyperactivity disorder. World J Clin Cases. 2023;11(14):3238–47. 37274030 10.12998/wjcc.v11.i14.3238 PMC10237121

[20] Sonuga-Barke EJ, Brandeis D, Cortese S, Daley D, Ferrin M, Holtmann M, Nonpharmacological interventions for ADHD: systematic review and meta-analyses of randomized controlled trials of dietary and psychological treatments. Am J Psychiatry. 2013;170(3):275–89. 23360949 10.1176/appi.ajp.2012.12070991

[21] Dentz A, Soelch CM, Fahim C, Torsello A, Parent V, Ponsioen A, Non-pharmacological treatment of Attention Deficit Disorder with or without Hyperactivity (ADHD). Overview and report of the first international symposium on the non-pharmacological management of ADHD. Encephale. 2024;50(3):309–28. 38326137 10.1016/j.encep.2023.04.010

[22] Wilens TE, Stone M, Lanni S, Berger A, Wilson RLH, Lydston M, Treating Executive Function in Youth With Attention Deficit Hyperactivity Disorder: A Review of Pharmacological and Non-Pharmacological Interventions. J Atten Disord. 2024;28(5):751–90. 38178649 10.1177/10870547231218925

[23] Lambez B, Harwood-Gross A, Golumbic EZ, Rassovsky Y. Non-pharmacological interventions for cognitive difficulties in ADHD: A systematic review and meta-analysis. J Psychiatr Res. 2020;120:40–55. 31629998 10.1016/j.jpsychires.2019.10.007

[24] Qiu H, Liang X, Wang P, Zhang H, Shum DHK. Efficacy of non-pharmacological interventions on executive functions in children and adolescents with ADHD: A systematic review and meta-analysis. Asian J Psychiatr. 2023;87:103692. 37450981 10.1016/j.ajp.2023.103692

[25] Van der Oord S, Prins PJ, Oosterlaan J, Emmelkamp PM. Efficacy of methylphenidate, psychosocial treatments and their combination in school-aged children with ADHD: a meta-analysis. Clin Psychol Rev. 2008;28(5):783–800. 18068284 10.1016/j.cpr.2007.10.007

[26] Arns M, Clark CR, Trullinger M, deBeus R, Mack M, Aniftos M. Neurofeedback and Attention-Deficit/Hyperactivity-Disorder (ADHD) in Children: Rating the Evidence and Proposed Guidelines. Appl Psychophysiol Biofeedback. 2020;45(2):39–48. 32206963 10.1007/s10484-020-09455-2 PMC7250955

[27] Cortese S, Ferrin M, Brandeis D, Holtmann M, Aggensteiner P, Daley D, Neurofeedback for Attention-Deficit/Hyperactivity Disorder: Meta-Analysis of Clinical and Neuropsychological Outcomes From Randomized Controlled Trials. J Am Acad Child Adolesc Psychiatry. 2016;55(6):444–55. 27238063 10.1016/j.jaac.2016.03.007

[28] Sun F, Fang Y, Chan CKM, Poon ETC, Chung LMY, Or PPL, Structured physical exercise interventions and children and adolescents with attention deficit hyperactivity disorder: A systematic review and meta-analysis. Child Care Health Dev. 2024;50(1):e13150. 37433667 10.1111/cch.13150

[29] Li D, Li L, Zang W, Wang D, Miao C, Li C, Effect of physical activity on attention in school-age children with ADHD: a systematic review and meta-analysis of randomized controlled trials. Front Physiol. 2023;14:1189443. 37576338 10.3389/fphys.2023.1189443 PMC10415683

[30] Zhang J, Diaz-Roman A, Cortese S. Meditation-based therapies for attention-deficit/hyperactivity disorder in children, adolescents and adults: a systematic review and meta-analysis. Evid Based Ment Health. 2018;21(3):87–94. 29991532 10.1136/ebmental-2018-300015 PMC10270372

[31] Flavell JH. Metacognition and cognitive monitoring: A new area of cognitive–developmental inquiry. Am Psychol. 1979;34(10):906–11.

[32] Schraw G, Dennison RS. Assessing Metacognitive Awareness. Contemp Educ Psychol. 1994;19(4):460–75.

[33] Baker L. Metacognition, comprehension monitoring, and the adult reader. Educ Psychol Rev. 1989;1(1):3–38.

[34] Fiorella L, Mayer RE. Eight Ways to Promote Generative Learning. Educ Psychol Rev. 2016;28(4):717–41.

[35] Bellon E, Fias W, De Smedt B. Metacognition across domains: Is the association between arithmetic and metacognitive monitoring domain-specific? PLoS One. 2020;15(3):e0229932. 32163453 10.1371/journal.pone.0229932 PMC7067420

[36] de Boer H, Donker AS, Kostons DDNM, van der Werf GPC. Long-term effects of metacognitive strategy instruction on student academic performance: A meta-analysis. Educ Res Rev. 2018;24:98–115.

[37] Ohtani K, Hisasaka T. Beyond intelligence: A meta-analytic review of the relationship among metacognition, intelligence, and academic performance. Metacogn Learn. 2018;13(2):179–212.

[38] Barnett SM, Ceci SJ. When and where do we apply what we learn? A taxonomy for far transfer. Psychol Bull. 2002;128(4):612–37. 12081085 10.1037/0033-2909.128.4.612

[39] Fonagy P, Moran GS, Edgcumbe R, Kennedy H, Target M. The roles of mental representations and mental processes in therapeutic action. Psychoanal Study Child. 1993;48:9–48. 8234562 10.1080/00797308.1993.11822377

[40] Cella M, Edwards C, Swan S, Elliot K, Reeder C, Wykes T. Exploring the effects of cognitive remediation on metacognition in people with schizophrenia. J Exp Psychopathol. 2019;10(2):2043808719826846.

[41] Gómez CM, Angulo-Ruiz BY, Rodríguez-Martínez EI, Ruiz-Martínez FJ, Muñoz EMP, Fernández MDL. Content and Process in the Brain. Implications for Clinical and Educational Approaches. In: Lopez-Soto T, Garcia-Lopez A, Salguero-Lamillar FJ, editors. The Theory of Mind Under Scrutiny: Psychopathology, Neuroscience, Philosophy of Mind and Artificial Intelligence. Cham (Switzerland): Springer Nature; 2023. p. 527–58.

[42] Willcutt EG, Doyle AE, Nigg JT, Faraone SV, Pennington BF. Validity of the executive function theory of attention-deficit/hyperactivity disorder: A meta-analytic review. Biol Psychiatry. 2005;57(11):1336–46. 15950006 10.1016/j.biopsych.2005.02.006

[43] Roberts BA, Martel MM, Nigg JT. Are There Executive Dysfunction Subtypes Within ADHD? J Atten Disord. 2017;21(4):284–93. 24214969 10.1177/1087054713510349 PMC4303540

[44] Karalunas SL, Nigg JT. Heterogeneity and subtyping in attention-deficit/hyperactivity disorder—Considerations for emerging research using person-centered computational approaches. Biol Psychiatry. 2020;88(1):103–10. 31924323 10.1016/j.biopsych.2019.11.002 PMC7210094

[45] Kofler MJ, Irwin LN, Soto EF, Groves NB, Harmon SL, Sarver DE. Executive Functioning Heterogeneity in Pediatric ADHD. J Abnorm Child Psychol. 2019;47(2):273–86. 29705926 10.1007/s10802-018-0438-2 PMC6204311

[46] Yue X, Liu L, Chen W, Preece DA, Liu Q, Li H, Affective-cognitive-behavioral heterogeneity of Attention-Deficit/Hyperactivity Disorder (ADHD): Emotional dysregulation as a sentinel symptom differentiating “ADHD-simplex” and “ADHD-complex” syndromes? J Affect Disord. 2022;307:133–41. 35367500 10.1016/j.jad.2022.03.065

[47] Mostert JC, Onnink AMH, Klein M, Dammers J, Harneit A, Schulten T, Cognitive heterogeneity in adult attention deficit/hyperactivity disorder: A systematic analysis of neuropsychological measurements. Eur Neuropsychopharmacol. 2015;25(11):2062–74. 26336867 10.1016/j.euroneuro.2015.08.010 PMC4788979

[48] Kofler MJ, Sarver DE, Spiegel JA, Day TN, Harmon SL, Wells EL. Heterogeneity in ADHD: Neurocognitive predictors of peer, family, and academic functioning. Child Neuropsychol. 2017;23(6):733–59. 27472007 10.1080/09297049.2016.1205010 PMC6083022

[49] García-Madruga JA, Elosúa MR, Gil L, Gómez-Veiga I, Vila JÓ, Orjales I, Reading Comprehension and Working Memory’s Executive Processes: An Intervention Study in Primary School Students. Read Res Q. 2013;48(2):155–74.

[50] Carretti B, Borella E, Elosúa MR, Gómez-Veiga I, García-Madruga JA. Improvements in Reading Comprehension Performance After a Training Program Focusing on Executive Processes of Working Memory. J Cogn Enhanc. 2017;1(3):268–79.

[51] Carretti B, Caldarola N, Tencati C, Cornoldi C. Improving reading comprehension in reading and listening settings: the effect of two training programmes focusing on metacognition and working memory. Br J Educ Psychol. 2014;84(2):194–210. 24829118 10.1111/bjep.12022

[52] Cornoldi C, Carretti B, Drusi S, Tencati C. Improving problem solving in primary school students: The effect of a training programme focusing on metacognition and working memory. Br J Educ Psychol. 2015;85(3):424–39. 26099785 10.1111/bjep.12083

[53] Capodieci A, Gola ML, Cornoldi C, Re AM. Effects of a working memory training program in preschoolers with symptoms of attention-deficit/hyperactivity disorder. J Clin Exp Neuropsychol. 2018;40(1):17–29. 28332914 10.1080/13803395.2017.1307946

[54] Capodieci A, Re AM, Fracca A, Borella E, Carretti B. The efficacy of a training that combines activities on working memory and metacognition: Transfer and maintenance effects in children with ADHD and typical development. J Clin Exp Neuropsychol. 2019;41(10):1074–87. 31401917 10.1080/13803395.2019.1651827

[55] Re AM, Capodieci A, Cornoldi C. Effect of training focused on executive functions (attention, inhibition, and working memory) in preschoolers exhibiting ADHD symptoms. Front Psychol. 2015;6:1161. 26300836 10.3389/fpsyg.2015.01161 PMC4526792

[56] Pisacco NMT, Sperafico YLS, Enricone JRB, Guimaraes LSP, Rohde LA, Dorneles BV. Metacognitive interventions in text production and working memory in students with ADHD. Psicol Reflex Crit. 2018;31(1):5. 32026089 10.1186/s41155-017-0081-9 PMC6966739

[57] Zheng H, Dong Y, Sun Y, Yang J, Yuan C, Wang J, Effectiveness of Metacognitive Regulation Intervention on Attention-Deficit-Hyperactivity Disorder Students’ Scientific Ability and Motivation. Front Psychol. 2021;12:747961. 35002845 10.3389/fpsyg.2021.747961 PMC8732764

[58] Kajka N, Kulik A. The Assessment of the Impact of Training With Various Metacognitive Interventions on the Enhancement of Verbal Fluency in School-Age Children With ADHD. J Atten Disord. 2023;27(1):89–97. 36129139 10.1177/10870547221121289

[59] Paananen M, Husberg H, Katajamaki H, Aro T. School-based group intervention in attention and executive functions: Intervention response and moderators. Front Psychol. 2022;13:975856. 36186366 10.3389/fpsyg.2022.975856 PMC9521625

[60] Tamm L, Epstein JN, Peugh JL, Nakonezny PA, Hughes CW. Preliminary data suggesting the efficacy of attention training for school-aged children with ADHD. Dev Cogn Neurosci. 2013;4:16–28. 23219490 10.1016/j.dcn.2012.11.004 PMC3617931

[61] Qian Y, Chen M, Shuai L, Cao QJ, Yang L, Wang YF. Effect of an Ecological Executive Skill Training Program for School-aged Children with Attention Deficit Hyperactivity Disorder: A Randomized Controlled Clinical Trial. Chin Med J. 2017;130(13):1513–20. 28639564 10.4103/0366-6999.208236 PMC5494912

[62] Kerns KA, Eso K, Thomson J. Investigation of a Direct Intervention for Improving Attention in Young Children With ADHD. Dev Neuropsychol. 1999;16(2):273–95.

[63] Dawson P, Guare R. Executive skills in children and adolescents: A practical guide to assessment and intervention. 2nd ed. New York (US): Guilford Press; 2010.

[64] Reina-Reina C, Antón E, Duñabeitia JA. A Systematic Literature Review of the Impact of Cognitive Stimulation Programs on Reading Skills in Children Aged between 6 and 12 Years Old. Educ Sci. 2024;14(3):229.

[65] Frisch C, Tirosh E, Rosenblum S. Children with ADHD Symptomatology: Does POET Improve Their Daily Routine Management? Children. 2023;10(6):1083. 37371314 10.3390/children10061083 PMC10297599

[66] Hahn-Markowitz J, Berger I, Manor I, Maeir A. Cognitive-Functional (Cog-Fun) Dyadic Intervention for Children with ADHD and Their Parents: Impact on Parenting Self-Efficacy. Phys Occup Ther Pediatr. 2018;38(4):444–56. 29494784 10.1080/01942638.2018.1441939

[67] Shah R, Sharma A, Grover S, Sachdeva D, Chakrabarti S, Avasthi A. Development and effectiveness of parent skills training intervention for Indian families having children with attention-deficit/hyperactivity disorder (ADHD). Asian J Psychiatr. 2021;64:102762. 34301518 10.1016/j.ajp.2021.102762

[68] Barkley RA. Defiant children: A clinician’s manual for assessment and parent training. 3rd ed. New York (US): Guilford Press; 2013.

[69] Chronis AM, Chacko A, Fabiano GA, Wymbs BT, Pelham WE. Enhancements to the behavioral parent training paradigm for families of children with ADHD: review and future directions. Clin Child Fam Psychol Rev. 2004;7(1):1–27. 15119686 10.1023/b:ccfp.0000020190.60808.a4

[70] Wells KC, Pelham WE, Kotkin RA, Hoza B, Abikoff HB, Abramowitz A, Psychosocial treatment strategies in the MTA study: rationale, methods, and critical issues in design and implementation. J Abnorm Child Psychol. 2000;28(6):483–505. 11104313 10.1023/a:1005174913412

[71] Coates J, Taylor JA, Sayal K. Parenting Interventions for ADHD: A Systematic Literature Review and Meta-Analysis. J Atten Disord. 2015;19(10):831–43. 24915737 10.1177/1087054714535952

[72] Tamm L, Nakonezny PA, Hughes CW. An open trial of a metacognitive executive function training for young children with ADHD. J Atten Disord. 2014;18(6):551–9. 22647287 10.1177/1087054712445782

[73] Solanto MV, Marks DJ, Wasserstein J, Mitchell K, Abikoff H, Alvir JM, Efficacy of meta-cognitive therapy for adult ADHD. Am J Psychiatry. 2010;167(8):958–68. 20231319 10.1176/appi.ajp.2009.09081123 PMC3633586

[74] Solanto MV, Surman CB, Alvir JMJ. The efficacy of cognitive-behavioral therapy for older adults with ADHD: a randomized controlled trial. Atten Defic Hyperact Disord. 2018;10(3):223–35. 29492784 10.1007/s12402-018-0253-1

[75] Solanto MV, Scheres A. Feasibility, Acceptability, and Effectiveness of a New Cognitive-Behavioral Intervention for College Students with ADHD. J Atten Disord. 2021;25(14):2068–82. 32880502 10.1177/1087054720951865

[76] Safren SA, Otto MW, Sprich S, Winett CL, Wilens TE, Biederman J. Cognitive-behavioral therapy for ADHD in medication-treated adults with continued symptoms. Behav Res Ther. 2005;43(7):831–42. 15896281 10.1016/j.brat.2004.07.001

[77] Korkman M, Kirk U, Kemp S. NEPSY: A developmental neuropsychological assessment: Manual. San Antonio (US): Harcourt Assessments; 1998.

[78] Wells A, Fisher P. Meta-Cognitive Therapy Without Metacognition: A Case of ADHD. Am J Psychiatry. 2011;168:327. 10.1176/appi.ajp.2010.10101467 21368310

[79] Moritz S, Lysaker PH, Hofmann SG, Hautzinger M. Going meta on metacognitive interventions. Expert Rev Neurother. 2018;18(10):739–41. 30232920 10.1080/14737175.2018.1520636

[80] Spencer TJ, Adler LA, Qiao M, Saylor KE, Brown TE, Holdnack JA, Validation of the adult ADHD investigator symptom rating scale (AISRS). J Atten Disord. 2010;14(1):57–68. 19794135 10.1177/1087054709347435

[81] Knouse LE, Safren SA. Current status of cognitive behavioral therapy for adult attention-deficit hyperactivity disorder. Psychiatr Clin North Am. 2010;33(3):497–509. 20599129 10.1016/j.psc.2010.04.001 PMC2909688

[82] Zylowska L, Ackerman DL, Yang MH, Futrell JL, Horton NL, Hale TS, Mindfulness meditation training in adults and adolescents with ADHD: a feasibility study. J Atten Disord. 2008;11(6):737–46. 18025249 10.1177/1087054707308502

[83] Bishop SR, Lau M, Shapiro S, Carlson L, Anderson ND, Carmody J, Mindfulness: A Proposed Operational Definition. Clin Psychol Sci Pract. 2004;11(3):230–41.

[84] Jha AP, Krompinger J, Baime MJ. Mindfulness training modifies subsystems of attention. Cogn Affect Behav Neurosci. 2007;7(2):109–19. 17672382 10.3758/cabn.7.2.109

[85] Lee CSC, Ng KH, Chan PCK, Peng X. Effectiveness of mindfulness parent training on parenting stress and children’s ADHD-related behaviors: A systematic review and meta-analysis. Hong Kong J Occup Ther. 2022;35(1):3–24. 35847187 10.1177/15691861211073826 PMC9279872

[86] Lee YC, Chen CR, Lin KC. Effects of Mindfulness-Based Interventions in Children and Adolescents with ADHD: A Systematic Review and Meta-Analysis of Randomized Controlled Trials. Int J Environ Res Public Health. 2022;19(22):15198. 36429915 10.3390/ijerph192215198 PMC9690476

[87] Phan ML, Renshaw TL, Caramanico J, Greeson JM, MacKenzie E, Atkinson-Diaz Z, Mindfulness-based school interventions: A systematic review of outcome evidence quality by study design. Mindfulness. 2022;13(7):1591–613. 36186722 10.1007/s12671-022-01885-9 PMC9524483

[88] Cairncross M, Miller CJ. The Effectiveness of Mindfulness-Based Therapies for ADHD: A Meta-Analytic Review. J Atten Disord. 2020;24(5):627–43. 26838555 10.1177/1087054715625301

[89] Evans S, Ling M, Hill B, Rinehart N, Austin D, Sciberras E. Systematic review of meditation-based interventions for children with ADHD. Eur Child Adolesc Psychiatry. 2018;27(1):9–27. 28547119 10.1007/s00787-017-1008-9

[90] Xue J, Zhang Y, Huang Y. A meta-analytic investigation of the impact of mindfulness-based interventions on ADHD symptoms. Medicine. 2019;98(23):e15957. 31169722 10.1097/MD.0000000000015957 PMC6571280

[91] Chimiklis AL, Dahl V, Spears AP, Goss K, Fogarty K, Chacko A. Yoga, Mindfulness, and Meditation Interventions for Youth with ADHD: Systematic Review and Meta-Analysis. J Child Fam Stud. 2018;27(10):3155–68.

[92] Follmer DJ, Sperling RA. The mediating role of metacognition in the relationship between executive function and self-regulated learning. Br J Educ Psychol. 2016;86(4):559–75. 27514725 10.1111/bjep.12123

[93] Turgut S, Uğurlu M. A moderated mediation analysis on the relationship between metacognition and mathematical resilience of gifted students. Psychol Schools. 2024;61(5):1848–67.

[94] Effeney G, Carroll A, Bahr N. Self-regulated learning and executive function: Exploring the relationships in a sample of adolescent males. Educ Psychol. 2013;33(7):773–96.

[95] Hofmann W, Schmeichel BJ, Baddeley AD. Executive functions and self-regulation. Trends Cogn Sci. 2012;16(3):174–80. 22336729 10.1016/j.tics.2012.01.006

[96] Slobodin O, Davidovitch M. Primary School Children’s Self-Reports of Attention Deficit Hyperactivity Disorder-Related Symptoms and Their Associations With Subjective and Objective Measures of Attention Deficit Hyperactivity Disorder. Front Hum Neurosci. 2022;16:806047. 35250516 10.3389/fnhum.2022.806047 PMC8888855

[97] Butzbach M, Fuermaier ABM, Aschenbrenner S, Weisbrod M, Tucha L, Tucha O. Metacognition in adult ADHD: subjective and objective perspectives on self-awareness of cognitive functioning. J Neural Transm. 2021;128(7):939–55. 33464422 10.1007/s00702-020-02293-w PMC8295131

[98] Pezzica S, Vezzani C, Pinto G. Metacognitive knowledge of attention in children with and without ADHD symptoms. Res Dev Disabil. 2018;83:142–52. 30205249 10.1016/j.ridd.2018.08.005

[99] Millenet S, Laucht M, Hohm E, Jennen-Steinmetz C, Hohmann S, Schmidt MH, Sex-specific trajectories of ADHD symptoms from adolescence to young adulthood. Eur Child Adolesc Psychiatry. 2018;27(8):1067–75. 29497857 10.1007/s00787-018-1129-9

[100] Tung I, Li JJ, Meza JI, Jezior KL, Kianmahd JS, Hentschel PG, Patterns of Comorbidity Among Girls With ADHD: A Meta-analysis. Pediatrics. 2016;138(4):e20160430. 27694280 10.1542/peds.2016-0430 PMC9923580

[101] Young S, Adamo N, Asgeirsdottir BB, Branney P, Beckett M, Colley W, Females with ADHD: An expert consensus statement taking a lifespan approach providing guidance for the identification and treatment of attention-deficit/hyperactivity disorder in girls and women. BMC Psychiatry. 2020;20(1):404. 32787804 10.1186/s12888-020-02707-9 PMC7422602

[102] Hinshaw SP, Nguyen PT, O’Grady SM, Rosenthal EA. Annual Research Review: Attention-deficit/hyperactivity disorder in girls and women: underrepresentation, longitudinal processes, and key directions. J Child Psychol Psychiatry. 2022;63(4):484–96. 34231220 10.1111/jcpp.13480

